# Neural Foundations Supporting Prosocial Behaviors: A Scoping Review of EEG/ERP Evidence During Adolescence and Emerging Adulthood

**DOI:** 10.1111/psyp.70340

**Published:** 2026-06-18

**Authors:** Rebecca Revilla, Cailee M. Nelson, Kate Flory, Sarah R. Edmunds, Abigail L. Hogan, Caitlin M. Hudac

**Affiliations:** ^1^ Department of Psychology University of South Carolina Columbia South Carolina USA; ^2^ Center for Autism and Neurodevelopment (CAN) Research Center University of South Carolina Columbia South Carolina USA; ^3^ Institute for Mind and Brain University of South Carolina Columbia South Carolina USA; ^4^ Department of Communication Sciences and Disorders, Arnold School of Public Health University of South Carolina Columbia South Carolina USA

**Keywords:** adolescence and emerging adulthood, ecological validity, electroencephalography (EEG), event related potentials (ERP), prosocial development, scoping review

## Abstract

Adolescence is a particularly important period for prosocial development given the foundational changes that occur in social and reward neural systems. Despite the temporal and developmental advantages of electroencephalography (EEG), no studies have compiled and analyzed existing EEG evidence of adolescent prosocial development. The current scoping review characterized how EEG methods are being used to study the rapid neural processes that underlie prosocial behaviors in early, middle, and late adolescence/emerging adulthood. Additionally, we evaluated the ecological validity and developmental appropriateness of the experimental paradigms included in this review and summarized neural correlates using a multidimensional developmental model. On September 13, 2025, two electronic databases were searched to identify studies that used traditional EEG methods to record state‐level prosocial behaviors in participants aged 10 to 25 years old. Thirty‐one studies were included in this review. Prosocial behavior themes measured across these studies included cooperation (32%), fairness (16%), giving (32%), and helping (19%). Studies commonly focused on college‐aged samples (94%) and evaluated event‐related potentials (81%), with three studies including additional spectral analyses (i.e., beta, delta, and theta). Two studies focused solely on spectral oscillations (i.e., beta, theta). P3 (84%) and FRN (55%) components were most frequently examined across reviewed studies and prosocial domains. Overall, results highlight a need for EEG studies to examine prosocial behaviors in earlier stages of adolescence, to consider the multidimensional nature of prosocial behaviors, and to begin evaluating prosocial behaviors that are more relevant to adolescents' day‐to‐day lives.

## Impact Statement

As the first scoping review of EEG studies that examined prosocial behaviors during adolescence and emerging adulthood, we provide an overview of how EEG methods have been used to examine prosocial behaviors past childhood. By systematically evaluating the ecological validity and developmental appropriateness of reviewed study paradigms, we provide guidance for future paradigm selection and creation. Lastly, our multidimensional organizational structure highlights gaps to be addressed in future EEG/ERP studies of adolescent prosocial behaviors.

## 1 Introduction

Prosocial behaviors, or actions performed with the intention of benefiting others (Thielmann and Pfattheicher 2022), are associated with positive societal and individual outcomes, including increased well‐being and fewer mental health difficulties (Hui et al. 2020; Memmott‐Elison et al. 2020). Prosocial behaviors include various social exchanges such as sharing, cooperating, giving, helping, and providing emotional support. A complex interplay between biological, cognitive, social, and situational factors influences the general development of prosocial behaviors (Eisenberg et al. 2015). Still, it is unclear how these factors interact to influence the development of specific prosocial behaviors, especially after early childhood (El Mallah 2020). Neurobiological processes underlie all human cognition and behaviors (Soon et al. 2008) and can be examined to help discern overlapping versus distinct cognitive mechanisms that drive and modulate specific prosocial behaviors across development (Wu and Hong 2022).

Substantial research has examined the neurobiological mechanisms underlying prosociality in infants and young children (Cowell et al. 2018; Pavlenko et al. 2023); however, there is a need to extend this characterization into adolescence due to the unique biological, cognitive, interpersonal, and contextual changes that occur during this period (El Mallah 2020; Galván 2021). For instance, adolescents experience increased independence from parents and heightened importance of peer affiliation (Eccles et al. 2003). As adolescents' social environment becomes more complex with their growing independence and new relationships to navigate, adolescents must engage in more advanced prosocial decision‐making than is required in early childhood (Eisenberg et al. 2015). For example, prosocial decision‐making in young childhood may involve sharing a toy with a sibling, whereas prosocial decision‐making in adolescence may involve risking one's social status to provide emotional support to a socially ostracized peer.

Adolescence is often considered a neurobiological transitional period between childhood and adulthood that is believed to start with puberty, as early as nine years old (Dorn et al. 2006). Hormones released during puberty correspond with neural reorganization, including synaptic proliferation, synaptic pruning, and axonal myelination (Blakemore and Choudhury 2006; Piekarski et al. 2017). Indeed, brain maturation and reorganization persist from the start of adolescence (~10–12 years) through the mid‐20s (Arain et al. 2013; Bethlehem et al. 2022; Giedd et al. 1999), which marks the approximate end of adolescence and a shift into emerging adulthood, though the exact timing of this shift depends upon individual differences in development (Arnett 2000; Sawyer et al. 2018).

Brain maturation trends suggest adolescence may be a sensitive period of development for higher‐order social processing, learning, and cognition, which guide prosocial behavior (Baker et al. 2025; Cheng et al. 2024; Crone and Dahl 2012; Larsen and Luna 2018). During adolescence, brain regions involved in affective processing (e.g., limbic system, striatum) and advanced social cognition (e.g., medial prefrontal cortex, temporoparietal junction) undergo substantial reorganization and specialization (Blakemore 2008; Crone and Dahl 2012). For instance, affective and reward‐related regions have shown heightened sensitivity to social and emotional cues during mid‐adolescence (~12–17 years), though specific age ranges vary by stimuli (van Leijenhorst et al. 2010; Pfeifer et al. 2011). In contrast, regions involved in advanced social cognition tend to mature through the mid‐20s (Blakemore 2008) while prolonged white matter development supports the strengthening connectivity between the prefrontal cortex and subcortical regions (Vijayakumar et al. 2021). Experience‐dependent neuroplasticity is also heightened through the mid‐20s, which increases adolescents' ability to adapt to and learn from positive and negative environmental input, particularly social information (Baker et al. 2025; Galván 2010).

Adolescent and adult brain imaging studies have demonstrated that brain mechanisms underlying prosocial behaviors involve multiple pathways, including socioaffective and sociocognitive neural regions (Crone et al. 2022; Wu and Hong 2022). Common functional brain networks are also observed in these mechanisms, including interactions between central executive, default mode, and salience networks that inform a value‐based system guided by the brain's reward areas (Sipes et al. 2022). In addition, the decision to act prosocially may have subsequent consequences on both the actor (e.g., positive feelings, self‐reward) and the target (e.g., relief, increased positive affiliation), which is proposed to reinforce these behaviors through a feedback loop (Wu and Hong 2022).

Still, there is an outstanding need to consider cognitive neuroscience approaches that can measure and describe the rapid neurocognitive processes underlying prosocial behaviors (Glover 2011). With millisecond‐level acquisition, electroencephalography (EEG) is optimal for precisely characterizing the moment‐to‐moment changes in neural processing with developmental continuity, from infancy to adulthood. EEG can also differentiate between automatic and controlled cognitive processes underlying prosocial behaviors (Hudac and Sommerville 2020; Johnson and de Haan 2015), which supplements existing knowledge of relevant brain regions and functional networks.

### 1.1 Existing Knowledge From EEG

One systematic review identified nine studies that examined differences elicited in event‐related potential (ERP) components (i.e., N2, Nc, P400, LPP, MFN, EPN) and EEG power spectra (i.e., frontal alpha asymmetry) corresponding with prosocial development in infants and children (Katus et al. 2020). Medial frontal negativity (MFN) components, including feedback‐related negativity (FRN), capture early automatic outcome processing and are believed to be more negative when individuals perceive outcomes that do not match their expectations (Martin and Potts 2011; Pfabigan et al. 2011). Cowell et al. (2019) examined the MFN in the anterior electrodes at 275–400 ms in 4‐ to 8‐year‐old children while they observed, as a third party, resources distributed equally, slightly unequally, and extremely unequally. They found increased negativity in the MFN for extremely unequal distributions compared with equal distributions. Early posterior negativity (EPN), typically seen around 150–350 ms in the temporo‐occipital region, reflects attention towards emotionally relevant stimuli and is more negative in response to such stimuli (Schupp et al. 2006). A study by Cowell and Decety (2015a) in 3‐ to 5‐year‐old children demonstrated greater negativity in the EPN when children viewed helping scenes compared to harming scenes. The N2, a negative‐going component generally peaking at approximately 180 ms, is related to attention allocation and conflict detection (Folstein and van Petten 2008) and was associated with children's neural responses to helping scenarios and painful stimuli. Specifically, the N2 was more negative in the frontal region when children viewed harming scenes compared to helping scenes (Cowell and Decety 2015a) and when children viewed painful stimuli compared to neutral stimuli (Cheng et al. 2014; Decety et al. 2018).

Similarly, the P400 and negative central (Nc) components, which capture attentional significance of stimuli and are linked with the P3 component in children and adults (Hajcak et al. 2010; Riggins and Scott 2020), were associated with infants' helping preferences. Gredebäck et al. (2015) found that the P400 was larger in the posterior temporal region when 6‐month‐old infants viewed helpers compared to hinderers, and Cowell and Decety (2015b) observed a larger Nc in the parietal region when 12‐ to 24‐month‐old infants/toddlers viewed helpers compared to hinderers. The late positive potential (LPP) component, often observed in the centro‐parietal region, is related to sustained attention towards a stimulus (DeCicco et al. 2012). Across several studies, the LPP was generally larger when infants and children viewed harm or extreme inequality (Cowell et al. 2019; Cowell and Decety 2015b; Decety et al. 2018; Meidenbauer, Cowell, and Decety 2018); however, one study found an opposite pattern in which the LPP was larger when children viewed helping scenes compared to harming scenes (Cowell and Decety 2015a). Lastly, this prior review found that infants' responses to helping scenarios and peer distress and children's responses to pain were distinct in EEG frontal alpha asymmetry (8–12 Hz), which is often associated with approach/withdrawal tendencies (Cowell and Decety 2015b; Crespo‐Llado et al. 2018; Decety et al. 2018; Smith et al. 2017). This is consistent with studies published after this review (Orekhova et al. 2020, Orekhova et al. 2023, Tan and Hamlin 2022).

While systematic reviews of EEG neural processes in infants and early childhood have demonstrated how EEG can be used to characterize neural processes underlying early prosocial development (Katus et al. 2020; Pavlenko et al. 2023), existing EEG evidence of adolescent prosocial development has not been compiled and analyzed. Unlike systematic reviews, scoping reviews provide a broad overview of available evidence in an area and provide insight into how research is being conducted on a topic as well as existing gaps (Munn et al. 2018; Peters et al. 2015). Given the unknown, potentially limited, state of EEG research examining prosocial behaviors during adolescence and the unique biological, cognitive, interpersonal, and contextual changes that occur during this period, we opted to conduct a scoping review to (1) characterize how EEG methods are being used to study state‐level prosocial behaviors (e.g., moment‐to‐moment decision making based on context) during adolescence, (2) summarize neural correlates in this area, and (3) identify gaps and future directions pertinent to development using EEG. Given our focus on neurobiological processes, we adhere to a definition of adolescence that extends from approximately 10–25 years of age (Sawyer et al. 2018); however, we refer to the latter end of adolescence (i.e., 18–25) as late adolescence/emerging adulthood in recognition of developmental shifts that occur during this period.

### 1.2 Methodological Considerations of the Current Review

Our framework for extracting and organizing relevant EEG methods and neural correlates of adolescent prosocial behaviors was based on findings from functional magnetic resonance imaging (fMRI) reviews and existing models of prosocial behaviors. Previous fMRI reviews (Crone et al. 2022; Sipes et al. 2022) of adolescent prosocial behaviors highlighted the frequent use of the Prisoner's Dilemma Task, Trust Game, Dictator Game, Public Goods Game, Ultimatum Game, Prosocial Cyberball Game, and variations of these games and other resource distribution tasks (see Table 1 for descriptions of common paradigms). These experimental paradigms present participants with opportunities to engage in prosocial decisions. Brain processes are examined in correspondence with participants' decisions and/or with their evaluations of decision outcomes. Despite the popularity of these paradigms, authors of both fMRI reviews acknowledged several limitations of these paradigms, such as a heavy reliance on a monetary decision‐making structure across paradigms, which may involve different cognitive processes than those used in adolescents' day‐to‐day prosocial decision‐making. In addition, many of these paradigms are complex because they were originally designed for adults. As a result, these paradigms may not be easily understood across adolescence.

**TABLE 1:** Descriptions of common prosocial paradigms.

Description	Example of participant view
**Prisoner's Dilemma Game** (Rilling et al. 2002): In this cooperation paradigm, participants see a matrix and take turns deciding whether to cooperate or deflect. Participants do not know what the other player decides on each turn until both players have decided. On each turn, participants win money based on their combined decisions. Participants win the greatest amount individually if they deflect when the other player cooperated, but the player who cooperated earns no money. Participants gain equal money if they both cooperate or both deflect; however, payment is higher when both cooperate than deflect. The **Chicken Game** (Fukui et al. 2006) is very similar to the Prisoner's Dilemma Game with the only difference being that both players deflecting leads to the worst outcomes for both players, either losing money or not gaining money	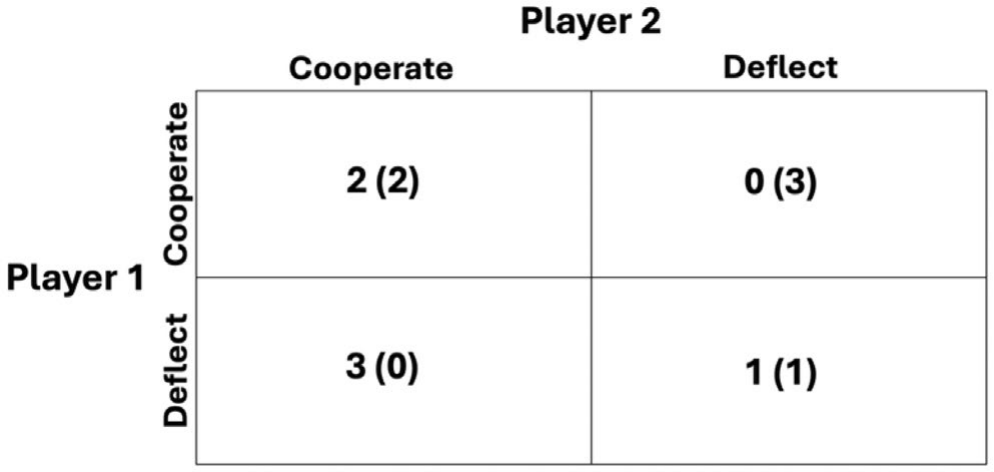
**Trust Game** (Sripada et al. 2009): In this trust paradigm, one player (investor) decides how much money to send to the other player (trustee). The money the investor sends to the trustee is multiplied. The trustee can keep all the money received or send a portion back to the investor	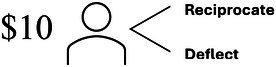
**Prosocial Cyberball Game** (van der Meulen et al. 2016): This ball‐tossing paradigm measures participant's inclusive ball‐tossing prosocial behaviors after the participant observes another player being excluded during the game	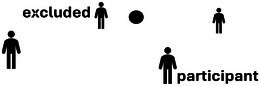
**Ultimatum Game** (Gabay et al. 2014): In this fairness paradigm, one player proposes a split of money between themselves and a second player. If the second player accepts the offer, they both receive the proposed money. If the second player rejects the offer, neither receive the proposed money	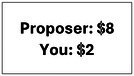
**Public Goods Game** (van Hoorn et al. 2016): In this cooperation paradigm, participants must decide how to split monetary tokens between themselves and a group of people. Tokens donated to the group of people are multiplied and divided equally amongst the group members	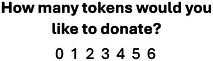

To our knowledge, a systematic method of evaluating prosocial paradigms in terms of ecological validity (i.e., how accurately paradigms reflect real‐world phenomena broadly) and developmental appropriateness (i.e., how well paradigms reflect adolescents' real‐life experiences and meet their developmental needs) has not been developed but has potential for guiding future cognitive neuroscience work. To begin addressing these limitations, we created a systematic method of extracting elements of ecological validity and assessing developmental appropriateness separately in early adolescence (10–13 years old), middle adolescence (14–17 years old), and late adolescence/emerging adulthood (18–25 years old). We used these metrics to characterize the ecological validity and developmental appropriateness of the paradigms included in this review.

Researchers also argue that it is imperative to consider the multidimensional nature of prosociality (Carlo and Randall 2002; El Mallah 2020; Memmott‐Elison et al. 2020). In other words, prosocial behaviors may change according to the circumstances of the specific situation; thus, the conditions of each situation should be characterized and analytically considered, when possible. To fully account for the multidimensional nature of prosocial behaviors, the current review characterizes and organizes the prosocial behaviors based upon the Heuristic Multidimensional Model of Prosocial Behaviors (Carlo and Padilla‐Walker 2020). In this way, we distinguished key dimensions of prosocial behaviors. First, the motive dimension of the model specifies whether the prosocial behavior is selflessly or selfishly motivated and intrinsically or extrinsically motivated. Second, the situation/context dimension describes characteristics of the situation/context that affect the behavior (i.e., dire or non‐dire, spontaneous or compliant, emotional or non‐emotional, and public or private). Third, the target dimension specifies characteristics of the behavior target, including kin or non‐kin, stranger or familiar other, and similar (in‐group) or dissimilar (out‐group). Importantly, this model posits that each dimension is embedded in culture, such that motives, situations, and targets influence prosocial behaviors differently depending on a person's cultural background.

## 2 Methods

### 2.1 Study Inclusion Criteria

To evaluate the full span of adolescence/emerging adulthood without extensive overlap into earlier childhood and adult periods, our review aimed to only include studies with participants aged 10–25 years old (Sawyer et al. 2018). Accordingly, studies were excluded from the current review if participant ages extended below 10 years old or above 25 years old (Rodrigues et al. 2015). For studies that did not report the range of participant ages, studies were only included if participants' mean age and standard deviation of age were within 10–25 years old. For instance, if a study reported a mean age of 24 years with a standard deviation of 3 years, that study was excluded. Studies of early childhood (Orekhova et al. 2023) and adults over 25 years old were excluded. Studies that did not report complete age information (e.g., mean without a standard deviation or range) were also excluded (Liu et al. 2022).

The current study aimed to characterize EEG/ERP neural mechanisms that underlie state‐level prosocial behaviors (e.g., moment‐to‐moment decision making based on context). Accordingly, studies were only included if EEG/ERP methods and prosocial behaviors were measured simultaneously. For clarity, this excludes papers focused on trait‐level prosocial characteristics, such as studies that linked resting state EEG with prosocial behaviors (Schiller et al. 2020), linked EEG/ERP with self‐report trait‐level prosocial measures (Lianekhammy and Werner‐Wilson 2015), or used passive viewing tasks that did not require a prosocial response (Orekhova et al. 2023).

Tasks were considered to measure a prosocial behavior if authors used common labels found in the prosocial literature to describe task specific behaviors (e.g., cooperation, giving, donation, altruism, prosocial, helping). It is important to note that “altruistic punishment” measured by experimental paradigms has been categorized by some researchers as a prosocial behavior (Ciaramidaro et al. 2018). We opted to exclude altruistic punishment from the current review given work establishing that punishment and altruism show no direct relation (Rodrigues et al. 2020).

This review only included studies of EEG/ERP methods using traditional analyses (i.e., ERP amplitude, ERP latency, and power spectra). Studies using advanced EEG methods, such as neural synchronization and source localization, were considered out of scope of the current review and subsequently excluded (Cho et al. 2020; Zhang et al. 2019). Lastly, studies were included if they were available in English and peer reviewed.

### 2.2 Search Strategy

The current review followed the PRISMA guidelines for scoping reviews (Tricco et al. 2018). The first author (R.R.) completed the following search, selection, data extraction, and coding procedures. An electronic database search of PsycInfo and Web of Science was conducted using the following search terms on September 13, 2025: (prosocial OR pro‐social OR altruis* OR coop* OR recipro* OR “trust game” OR “helping behav*” OR help* OR shar* OR giv* OR comfort* OR donat* OR defend*) AND (adolescen* OR teen* OR youth OR “late childhood” OR “emerging adult*” OR “early adult*” OR “young adult*” OR college) AND (EEG OR electroencephalography OR electrophysiology OR “event‐related potentials” OR “evoked‐response potentials”).

### 2.3 Selection Process

**FIGURE 1:**
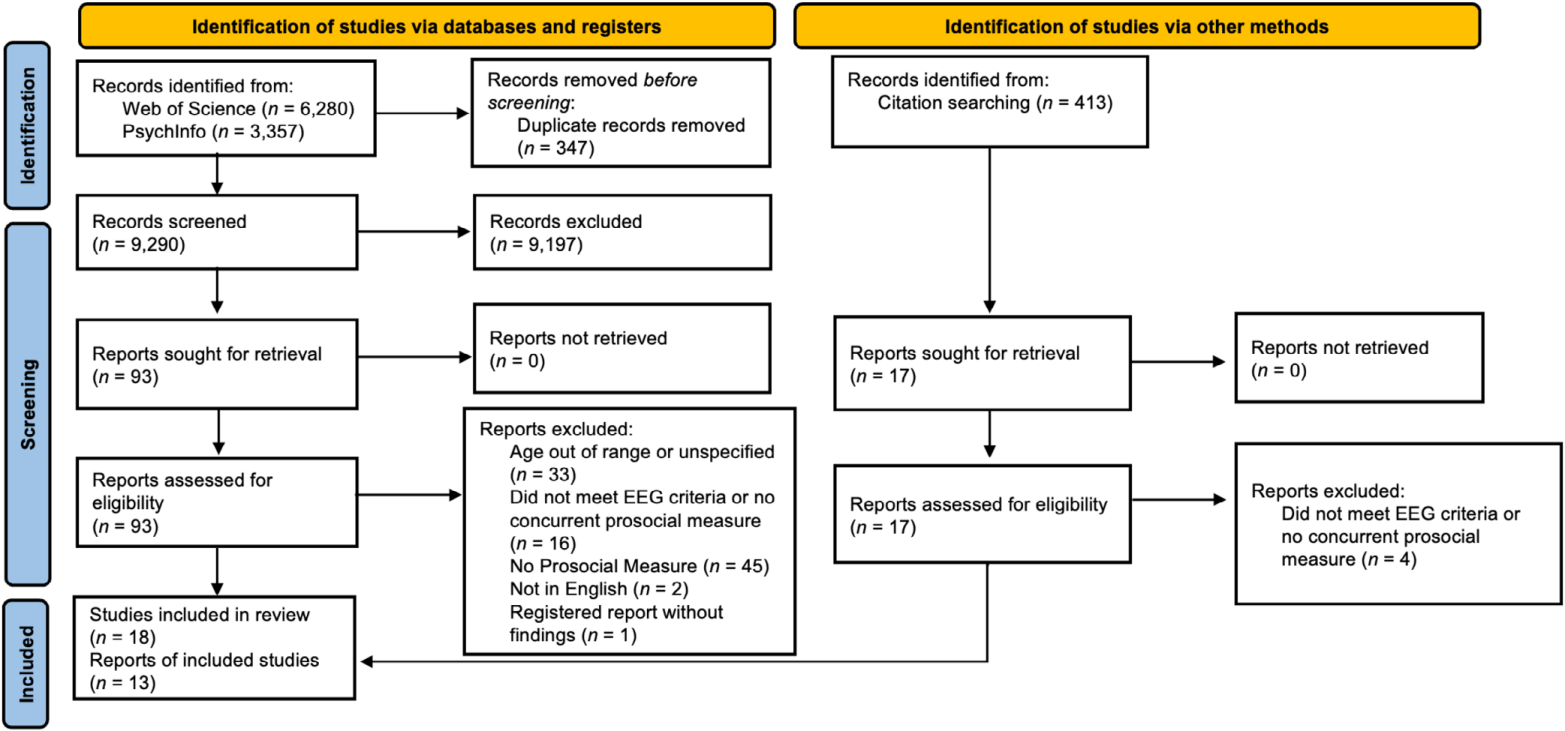
PRISMA diagram.

Search results produced 9637 studies. After removing duplicates between the databases, 9290 studies remained. All screening procedures were initially conducted by the first author (R.R.). Titles and abstracts were screened for characteristics that met exclusion criteria, including no use of EEG/ERP methodology, a lack of prosocial constructs, and samples with ages outside the pre‐established age range. Ninety‐three studies were identified as potentially relevant to the current review. Full texts of these studies were reviewed. Eighteen studies met inclusion criteria, and 75 studies were removed from the final review due to being out of the specified age range (*n* = 33), not meeting EEG inclusion criteria (*n* = 16), not having a measure of prosocial behavior (*n* = 45), being written in a language other than English (*n* = 2), and being a registered report without results (*n* = 1). References cited by these 18 studies were also reviewed manually using Google Scholar to identify recently published studies that may have been missed in the initial search. An additional 13 studies were identified from this manual search. In total, 31 studies were included in this review. This process is depicted in Figure 1. A second author (C.M.N.) conducted a follow‐up independent review of the 9290 study titles and abstracts produced by the original database search to ensure all eligible papers were included in this review. All studies identified as potentially relevant in this second screening had already been included in the final study sample.

### 2.4 Data Extraction and Coding Process

#### 2.4.1 Prosocial Behaviors

**FIGURE 2:**
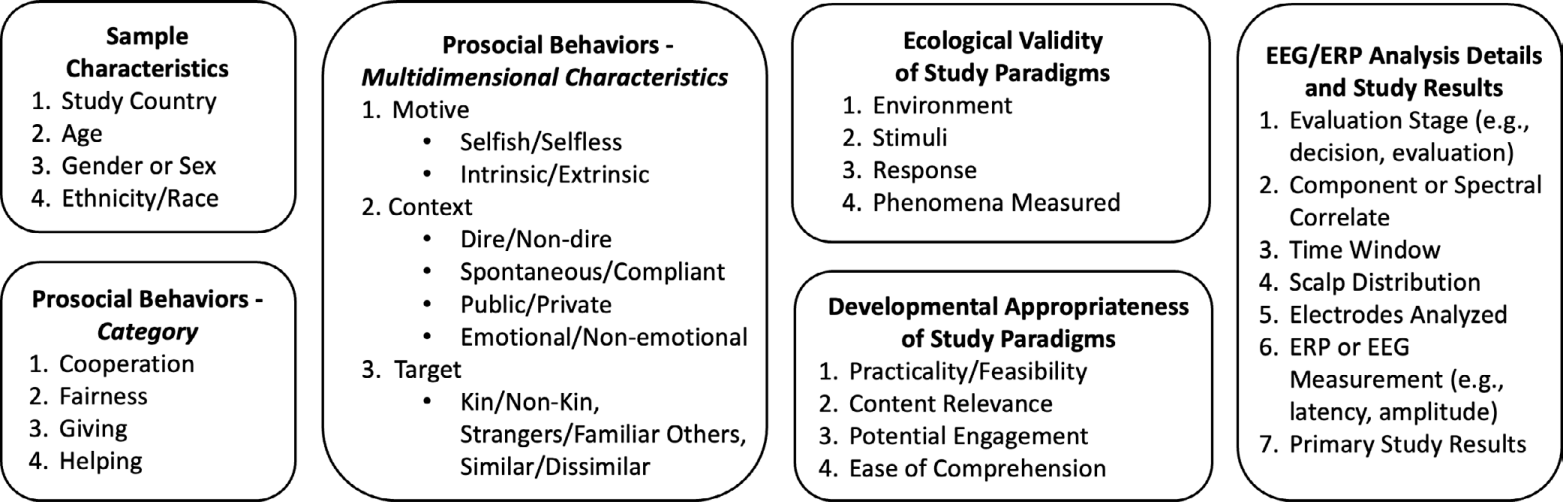
Data extraction and coding process. Data was extracted and coded according to sample characteristics (Table 2), EEG/ERP analysis details and study results (SITable1.Results tab in Data S1), prosocial behavior group (see Category columns in searchable Supporting Information database), multidimensional characteristics of prosocial behaviors (SITable2.Task tab in Data S1), ecological validity of study paradigms (SITable3.EV tab in Data S1), and developmental appropriateness of study paradigms (SITable4.DA tab in Data S1).

For each study, specific prosocial behaviors were labeled based on language used in the study text and the phenomenon being measured by each paradigm (Figure 2). Characteristics of prosocial behaviors were extracted and organized by first author (R.R.) according to the Heuristic Multidimensional Model of Prosocial Behaviors (Carlo and Padilla‐Walker 2020) and based upon three primary dimensions: (1) *Motive* (i.e., selflessly/selfishly, intrinsically/extrinsically), (2) *Situation/Context* (i.e., dire/non‐dire, spontaneous/compliant, emotional/non‐emotional, and public/private), and (3) *Target* (i.e., kin/non‐kin, strangers/familiar others, and similar/dissimilar).

#### 2.4.2 EEG/ERP Correlates

Details for ERP components and EEG spectral analyses were extracted by first author (R.R.) from each study. This included: (1) *Evaluation Stage* (e.g., *time‐locked ERP*), (2) *Component/Spectral Correlate*, (3) *Time‐Window*, (4) *Scalp Distribution*, and (5) *EEG/ERP Measurement* (e.g., latency, amplitude, power spectra). Primary EEG/ERP findings were also extracted from each study and summarized.

#### 2.4.3 Ecological Validity

Criteria for this measure was developed based on three dimensions of ecological validity (i.e., environment, stimuli, response) defined by Schmuckler (2001). As outlined in SI Figure 3 in Data S2: (1) *Environment* criteria evaluates the characteristics of the setting in which the study was conducted, including how closely the setting reflects the environment the phenomenon naturally occurs in and with whom interactions occur; (2) *Stimuli* criteria evaluates the visual representation of the target activity and how closely it reflects real‐world phenomena; (3) *Response* criteria evaluates the method of recording participants' behavioral responses, specifically how closely study responses reflect natural responses that would occur with the target phenomenon; and (4) *Phenomena Measured* (added dimension for this scoping review) evaluates whether the activity captures prosocial behaviors without reliance on monetary structure. As mentioned previously, paradigms measuring prosocial behaviors in adolescents appear to rely heavily on a monetary decision‐making structure, which likely involves different cognitive processes than prosocial decision‐making that does not rely on a monetary structure.

Ecological validity of experimental paradigms was independently evaluated by two authors (R.R., C.M.N.) across the four criteria described above with strong agreement across independent coders for all criteria (see SI Figure 1 in Data S2). Reviewers discussed their independent scoring and amended discrepant scoring after discussion. To create a summary ecological validity score for each paradigm, scores were tallied across the four criteria and summed. Of 11 total possible points, final scores of 0–3 were categorized as low validity, scores of 4–7 were categorized as moderate validity, and scores of 8–11 were categorized as high validity. Overall, higher points indicated greater ecological validity across elements.

#### 2.4.4 Developmental Appropriateness

At the onset of the scoping review, we identified four elements for evaluating the developmental appropriateness of experimental paradigms (SI Figure 4 in Data S2). *Feasibility* was evaluated based on the range of skills the target population (i.e., typically developing adolescents with average abilities) would likely need to successfully complete the task, such as motor skills, processing speed, and cognitive abilities. *Content Relevance* was evaluated based on the likelihood that the target population would do the activity captured in the paradigm in their daily life. *Engagement Potential* considered how engaging or interesting the paradigm would be for the target population. *Ease of Comprehension* evaluated the likelihood that the target population would understand how to perform the given task based on the task instructions and design.

Each paradigm was independently rated across the four elements by two authors (R.R., C.M.N.) for its potential to be used in early (10–13 years old), middle (14–17 years old), and late (18–25 years old) adolescence/emerging adulthood (Arnett 2000; Eccles et al. 2003; Sawyer et al. 2018). Reviewers discussed their independent scoring and amended discrepant scoring after discussion. Generally, inter‐rater reliability (see SI Figure 2 in Data S2) was moderate to high for most categories with two exceptions—early adolescence feasibility had poor agreement and reliability metrics that indicated a coding inconsistency, and late adolescence/emerging adulthood engagement potential had weak reliability. As we describe in the limitations section of the discussion, we recommend that future studies refine the coding scale to avoid imbalanced marginal distributions.

Points were allocated based on whether experimental paradigms satisfied the criteria of each element. Reviewers discussed their independent scoring and amended discrepant scoring after discussion. Scores were then tallied across elements and summed to create a summary developmental appropriateness score for each paradigm. Of a total 12 points, scores of 0–3 were categorized as low, scores of 4–7 were categorized as moderate, and scores of 8–12 were categorized as high.

## 3 Results

### 3.1 Demographic Characteristics

Demographic characteristics are presented in Table 2. Study sample sizes ranged from 14 to 82 participants. Most studies reported a relatively even distribution across sex (i.e., male, female) and gender (i.e., man, woman). One study reported an all‐male sample (Kanso et al. 2014). Most studies were conducted in China (*n* = 26). Other countries included Canada (*n* = 1), Chile (*n* = 1), the United Kingdom (*n* = 1), and the United States (*n* = 2). Of the 31 studies included in this review, 29 focused on undergraduate student samples, and only two studies were developmentally focused and included early to middle adolescent samples (Kwak et al. 2020; Meidenbauer, Cowell, Killen, and Decety 2018). Specifically, all studies examining cooperation and helping used undergraduate student samples, while one study examining fairness (Meidenbauer, Cowell, Killen, and Decety 2018) and one study examining giving (Kwak et al. 2020) included early to middle adolescent samples. Race/ethnicity and socioeconomic status, as measured by household income, were only reported by one study (Meidenbauer, Cowell, Killen, and Decety 2018).

**TABLE 2:** Summary of sample characteristics derived from scoping review.

Author	Country	*N*	Age M (SD/SE) and/or range	% Female/woman^a^	Ethnicity/race	EV total score	DA total score
Carlson et al. (2016)	Canada	20	20.6 (3.73)	70	Not provided	1	12
Chen et al. (2017)	China	14	22.3; 20–25	57	Not provided	3	5
Cui et al. (2018)	China	34	21.33 (0.33)	32	Not provided	1	4
Gan et al. (2016)	China	24	20.6 (1.79)	50^a^	Not provided	1	12
Gan et al. (2022)	China	41	19.6 (1.36)	49^a^	Not provided	1	12
Kanso et al. (2014)	United Kingdom	28	18–21	0	Not provided	2	9
Kwak et al. (2020)	United States	62	10–17	62	Not provided	1	7
Lavín et al. (2023)	Chile	32	21.4 (1.3)	41^a^	Not provided	1	5
Li et al. (2020)	China	32	20.14 (1.99)	50^a^	Not provided	3	4
Li, Li, et al. (2021)	China	26	21.22 (2.17)	62^a^	Not provided	2	9
Li, Xu, and Zhong (2021)	China	30	21.23 (2.14)	53^a^	Not provided	2	5
Li et al. (2022)	China	30	20.56 (2.11)	53^a^	Not provided	2	4
Li et al. (2025)	China	76	21.08 (0.35)	47	Not provided	1	5
Ma et al. (2015)	China	18	22.17 (1.92)	33	Not provided	1	7
Meidenbauer, Cowell, Killen, and Decety (2018)	United States	82	11.6 (2.62); 11–16	55	39% European American, 34% African American, 10% Latino, 4% Asian, 13% multiracial	1	10
Pei et al. (2021)	China	17	23.17 (1.58)	53	Not provided	4	5
Peng et al. (2021)	China	37	20.6 (1.85)	54^a^	Not provided	2	4
Peng et al. (2025)	China	48	20.04 (1.88)	50^a^	Not provided	2	4
Tan et al. (2022)	China	31	19.45 (1.20)	52	Not provided	1	6
Tan et al. (2025)	China	33	18.82 (1.07)	67	Not provided	1	7
Teng et al. (2018)	China	68	21.50 (1.88)	54	Not provided	1	7
Wang et al. (2013)	China	15	22.7; 18–25	47	Not provided	4	4
Wang, Zhu, et al. (2022)	China	24	20.79 (2.00)	50	Not provided	2	5
Wang, Fu, et al. (2022)	China	20	20.05 (0.44)	45	Not provided	2	4
Xu et al. (2023)	China	23	18–25	61	Not provided	1	9
Yang et al. (2022)	China	72	20.1 (1.58)	53	Not provided	2	6
Ye et al. (2022)	China	29	19.74 (1.85)	59	Not provided	1	12
Yuan et al. (2019)	China	34	22.10 (1.66)	41	Not provided	3	4
Zhan et al. (2018)	China	23	22.15 (0.88)	52	Not provided	1	9
Zhang et al. (2019)	China	70	20.4 (2.2)	49	Not provided	3	3
Zhang et al. (2021)	China	27	21.46 (1.55)	56	Not provided	1	9

Abbreviations: DA, developmental appropriateness; EV, ecological validity; M, mean; *N*, sample size; SD, standard deviation.

^a^ Study reported gender, not sex.

### 3.2 EEG/ERP Correlates

Studies were sorted into one of four broad prosocial behavior domains based on language used in the study text and the phenomenon being measured by each paradigm, including cooperation (*n* = 10, 32.3%), fairness (*n* = 5, 16.1%), giving (*n* = 10, 32.3%), and helping (*n* = 6, 19.4%). Cooperation paradigms included tasks that required participants to coordinate their actions with another individual towards a shared outcome or goal. Fairness paradigms encompassed tasks that involved evaluating and/or enforcing equitable resource distribution, including resource allocation and ultimatum game paradigms. Giving paradigms involved one‐sided transfers of participants' own resources to another person or entity (e.g., charity). Helping paradigms included tasks in which participants could provide or imagine how they would provide assistance to another individual. Paradigm characteristics for each study are summarized in a searchable Supporting Information database (SITable2.Task in Data S1).

EEG and ERP correlates for each prosocial behavior are also summarized in the searchable Supporting Information database (SITable1.Results in Data S1). For ease of interpretation, the P3/P300 and its subcomponents (P3a, P3b) will herein be discussed as P3, and the P2/P230 will be discussed as P2. EEG and ERP correlates were grouped by subdomains of prosocial behavior. We also extracted the experimental stage when these correlates were examined. We identified five evaluation stages that were examined across studies, including participants' (1) evaluation of another's action that impacts the participant (*n* = 11, 35.5%), (2) implicit attention during forced prosocial interaction (*n* = 1, 3.2%), (3) evaluation of another's action that impacts a third party (*n* = 2, 6.5%), (4) evaluation of context prior to a decision (*n* = 12, 38.7%), and (5) evaluation of decision outcomes (*n* = 7, 22.6%). Most studies examined ERPs (81%) with three of these studies including additional spectral analyses (i.e., beta, delta, and theta). Only two studies focused solely on spectral oscillations (i.e., beta, theta).

#### 3.2.1 Cooperation

Ten studies used cooperation paradigms; seven used the Chicken Game, one used a modified Prisoner's Dilemma Game, one used a modified Gambling Task, and one used an Eriksen Flanker Paradigm. The Chicken Game and Prisoner's Dilemma Game have a similar premise to evaluate participants' trial‐by‐trial decisions to cooperate with or aggress against another participant to gain monetary rewards. In the Prisoner's Dilemma Game (Rilling et al. 2002), each player decides whether to cooperate or deflect with a second player in a series of rounds. Players do not know what the other player decides in each round until both players have decided. Players gain money based on players' combined decisions. For instance, players gain equal money if they both cooperate or both deflect; however, the amount of money is greater when both cooperate versus deflect. A player gains the greatest amount individually if they deflect when the other player cooperated; however, the player who cooperated gains no money. The Chicken Game (Fukui et al. 2006) is very similar to the Prisoner's Dilemma Game with the only difference being that both players deflecting leads to the worst monetary outcomes for both players. In contrast to the Chicken Game and Prisoner's Dilemma paradigms, the Eriksen Flanker Paradigm and the Modified Gambling Task require participants to work together to earn joint monetary rewards in a cooperation condition and to compete against each other in a competition condition.

Across cooperation studies, the FRN (*n* = 8, 80%) and P3 (*n* = 9, 90%) components were most frequently examined. Notably, most studies focused on ERPs time‐locked to when participants observed their opponent's decision, rather or in addition to being time‐locked to the participant's own decision. FRN modulations while playing the Chicken Game and the Prisoner's Dilemma Game showed areas of overlap and discrepancy. Four studies found that when participants observed their opponent's decision, FRN amplitudes were more negative for aggressive decisions compared to cooperative decisions (Chen et al. 2017; Peng et al. 2025; Yuan et al. 2019; Zhang et al. 2019). This effect was consistent whether opponents were strangers or participants' friends (Chen et al. 2017). When the FRN was time‐locked to the participant's own decision, effects were inconsistent. Specifically, Peng et al. (2025) found the FRN was more negative before participants chose to aggress versus cooperate, whereas Yuan et al. (2019) did not find a significant effect.

When analyses examined the interaction between participants' own choices and their opponents' choices in the back‐and‐forth exchange, difference FRN (dFRN) amplitudes showed more variable patterns across studies. Wang et al. (2013) found the largest difference in FRN amplitude (subtracting gain trials from loss trials) when participants cooperated then subsequently observed their opponent aggress. Peng et al. (2021) used a different strategy and computed the dFRN based upon participants' FRN response to their opponent's choice (subtracting average FRN response during opponent cooperation trials from average FRN response during opponent aggression trials). Based on this calculation, a more negative dFRN indicates more sensitivity to opponents' decisions to aggress. Further, the dFRN was calculated separately for trials in which participants chose to aggress and trials in which participants chose to cooperate. The dFRN in trials that participants chose to aggress was more negative than trials in which participants chose to cooperate. Although Li, Xu, and Zhong (2021) also computed the dFRN based upon opponent choice, they found results more similar to Wang et al. (2013)—a more negative dFRN amplitude when participants cooperated then subsequently observed their opponent aggress in both monetary and non‐monetary incentivized trials. However, for monetary incentivized trials only, a more negative dFRN amplitude was recorded when participants aggressed then subsequently observed their opponent cooperate. Additionally, a more negative dFRN amplitude was recorded in monetary incentivized trials versus non‐monetary incentivized trials (Li, Xu, and Zhong 2021).

One study reported sex differences for the FRN while performing a Modified Gambling Task in cooperative and competitive conditions (Yang et al. 2022). For males, larger versus smaller rewards elicited more negative FRN amplitudes in both cooperative and competitive contexts. For females, this effect was observed in the cooperative condition but not the competitive condition. Additionally, for males only, more negative FRN amplitudes were elicited across both contexts when their opponent experienced a loss versus a gain and in the cooperative condition when participants received a larger reward versus smaller reward.

Six studies reported more positive P3 amplitudes when participants observed opponents' decisions to cooperate versus aggress (Chen et al. 2017; Li, Xu, and Zhong 2021; Peng et al. 2025; Wang et al. 2013; Yuan et al. 2019; Zhang et al. 2019). Several studies also examined how the P3 varied based upon opponent characteristics. For instance, P3 amplitudes were more positive when participants observed relatively cooperative opponents aggress rather than cooperate (Peng et al. 2021) and when participants made reciprocal cooperative choices with a stranger but not a friend (Chen et al. 2017). The P3 was also sensitive to participant sex differences such that it was more positive for male participants when they observed their opponent gain rewards and female participants when they observed their opponent lose rewards (Yang et al. 2022). Regarding the role of the P3 in participant's own behavior, Wang et al. (2013) found a more positive P3 after participants made aggressive versus cooperative decisions. Additionally, Kanso et al. (2014) demonstrated that participants in positions of power elicited less positive P3 amplitudes while engaging in a cooperative attention task with a subordinate opponent.

The N1 (an index of initial attention; Parasuraman 1980) and LPP were studied less frequently, each appearing in only one study (Zhang et al. 2019). Here, N1 amplitude was more negative for cooperative versus aggressive opponent feedback, and LPP amplitude was more positive when participants planned to reciprocate their opponent's cooperation. Additionally, only three studies investigated modulation of neural oscillations during cooperation paradigms, primarily focusing on theta (an index of error monitoring; Sandre and Weinberg 2019) and beta (an index of inhibitory control; Tempel et al. 2020) frequencies. Theta power was increased when participants observed relatively cooperative opponents aggress (Peng et al. 2021) and had different associations with participants' cooperation rates depending on monetary context (Peng et al. 2025). Differences in theta and beta power also predicted participants' subsequent aggressive and cooperative behaviors (Wang, Fu, et al. 2022).

#### 3.2.2 Fairness

Of the five studies measuring fairness, four used an adapted Ultimatum Game and one used a Resource Allocation Game. In the Ultimatum Game, one of two players is given the role of the proposer who must split a sum of money between themselves and the other player. The other player then decides to accept or reject the offer. If the offer is accepted, both players receive the proposed sum, but if the offer is rejected, neither receives rewards. The Resource Allocation Game evaluated participants' distributions of resources between a setting with economic need and a setting without economic need.

Studies utilizing the Ultimatum Game examined ERPs when participants evaluated proposed monetary splits and aimed to explain how characteristics of the proposer influence participants' behavioral and neural responses. Participants were more likely to accept unfair offers from proposers with low wealth (Pei et al. 2021; Wang, Zhu, et al. 2022) and good intentions (Ma et al. 2015). Participants' behavioral patterns aligned with ERP results such that FRN amplitude was more negative when participants received unfair offers from proposers with bad intentions (Ma et al. 2015) and high to moderate wealth (Pei et al. 2021; Wang, Zhu, et al. 2022). Additionally, P3 amplitude was more positive when participants received unfair offers from proposers with good intentions (Ma et al. 2015) and moderate wealth (Wang, Zhu, et al. 2022). The LFN (late frontal negativity), a negative‐going component related to increased conflict (Polezzi et al. 2008), was more negative when participants received fair offers from low wealth proposers and unfair offers from high wealth proposers (Pei et al. 2021). One study uniquely evaluated ERP (i.e., P2, P3, MFN) and spectral differences (i.e., delta, theta, beta) associated with opportunity versus outcome distribution equity (Li et al. 2025).

Meidenbauer, Cowell, Killen, and Decety (2018) used a Resource Allocation Game to explore early (i.e., P2), middle (i.e., P3), and late positive‐going components (i.e., LPP) implicated in evaluating distributions of resources between two settings with and without economic need. In 8‐ to 10‐year‐olds, P2 amplitude, linked with attention allocation and sensory processing (Potts 2004), was more positive when participants viewed equal distributions of resources regardless of settings' economic need. In 11‐ to 16‐year‐olds, P3 amplitude was more positive when participants viewed all resources or no resources distributed to settings with economic need. Across youth, LPP amplitude was most positive when all resources were distributed to settings with economic need. ERP modulations while viewing distributions predicted how positively youth rated resource distributions, youths' personal distribution decisions on a subsequent task where they decided how to allocate resources, and their time spent playing an online charity game.

#### 3.2.3 Giving

Of the 10 studies that examined giving, five used variations of donation tasks, three used gambling tasks, one used a Coin‐Guess Task, and one used an adapted Dictator Game. The donation tasks required participants to accept or reject donation offers to charities across numerous trials, though the attributes and designs across these tasks varied greatly. In the three gambling tasks, participants' decisions led to monetary rewards and losses for themselves and another entity (e.g., charity, friend). Similarly, the coin‐guess task required participants to guess and report the outcomes of coin tosses to win monetary rewards for themselves or various charities. However, this task uniquely examined altruistic deception and had a condition in which participants could provide deceptive responses for more rewards. The adapted Dictator Game required participants to make a series of decisions that resulted in more, less, or equal money for themselves and a another (i.e., future player, charity).

When participants were making donation decisions, the P2, N2, and P3 components were most frequently examined. P2 amplitude was more positive during conditions requiring fast versus slow donation decisions (Carlson et al. 2016), in money‐primed conditions (Li, Li, et al. 2021), when making donation decisions aligned with larger versus smaller groups of online peer donors (Ye et al. 2022), and when making voluntary versus mandatory donations (Zhang et al. 2021). N2 amplitude was more negative when participants made high‐to‐moderate cost versus low‐cost donations (Li, Li, et al. 2021), when making donation decisions that aligned with smaller versus larger groups of online peer donors (Ye et al. 2022), and when participants had an opportunity to provide deceptive responses to receive more rewards for themselves versus charity (Cui et al. 2018). P3 amplitude was more positive when participants had to make fast donation decisions to charities they highly empathized with (Carlson et al. 2016), made donation decisions aligning with larger versus smaller groups of online peer donors (Ye et al. 2022), made high‐cost donations in money‐primed conditions (Li, Li, et al. 2021), and made donation decisions after viewing social information that showed other people made higher donations than the participant (Xu et al. 2023). One study found P3 sensitivity predicted participants' subsequent donation behaviors (Carlson et al. 2016).

The LPP, FRN, and theta were also examined while participants were making donation decisions. LPP amplitude was more positive when participants voluntarily made donations versus when participants were mandated to make donations (Zhang et al. 2021). A more negative FRN amplitude was observed when participants made donation decisions after viewing social information that showed other people made higher donations than the participant (Xu et al. 2023). Theta activity was increased when participants decided between options that provided low pay to self and low pay to a charity versus low pay to self and high pay to a charity (Lavín et al. 2023). Increased theta activity was also associated with more prosocial decisions (Lavín et al. 2023).

The P2, P3, and FRN components were examined while participants evaluated decision outcomes, including losses and gains for themselves and another entity (e.g., charity). A more positive P3 amplitude was elicited when participants viewed their own gains and losses versus gains and losses for charity (Tan et al. 2022). The level of empathy participants felt for charities modulated the P3, such that P3 amplitude was most positive for decisions that would impact themselves and more positive for decisions that would impact high‐empathy charities than low‐empathy charities (Tan et al. 2025). A more negative FRN amplitude was elicited when participants viewed wins and losses for charity versus their own wins and losses (Tan et al. 2022). This effect was also modulated by the level of empathy participants felt for charities, such that the FRN was most negative for decisions impacting low‐empathy charities and more negative for decisions impacting high‐empathy charities than oneself (Tan et al. 2025).

A study by Kwak et al. (2020) compared P2, P3, and FRN differences between 10 and 17‐year‐old adolescents' and young adults' responses to winning or losing monetary rewards for themselves or a friend. P2 amplitude was more positive in adolescents when they viewed their own monetary losses versus a friend's losses and in adults when they viewed any monetary wins, including for themselves and a friend. For adults, FRN was more negative when they viewed their own monetary losses versus wins; however, the FRN was deemed too weak to statistically explore in adolescents. The P3 was examined at two time periods. At 300–375 ms, P3 amplitude was more positive for adults and adolescents when they viewed monetary wins versus losses. In contrast, at 425–800 ms, P3 amplitude was more positive for adults when they viewed monetary losses versus wins but equal for adolescents across these conditions. At 300–375 and 425–800 ms, P3 amplitude was more positive when adults viewed self‐related outcomes versus friend‐related outcomes but equal for adolescents across these conditions.

#### 3.2.4 Helping

Of the six helping studies, three studies used helping decision tasks, two used lottery‐choosing tasks, and one used a Dilemma Scenario Priming Paradigm. In the helping decision tasks, participants read stories describing a person in need of assistance and decided whether they would provide help. In two versions of this task, participants also received feedback indicating whether their decisions to help were successful. In the lottery‐choosing tasks, participants made decisions to give up portions of their own money to help a friend or a stranger gain money or avoid losing money. Similarly, in the Dilemma Scenario Priming Paradigm, participants were given moral dilemmas involving a stranger, an acquaintance, and a friend and had to decide between preventing harm or maintaining personal benefits.

N2, P3, and LPP components were examined while participants considered situational context to make decisions to help various targets. More negative N2 amplitudes were elicited when participants were deciding to give up their own money to prevent loss for a stranger and a friend (Li et al. 2020). In a follow‐up study comparing private and public decision‐making, a more negative N2 amplitude was elicited when participants were privately versus publicly deciding to help acquaintances and strangers (Li et al. 2022). However, when deciding to help friends, no difference in N2 amplitude was found for private versus public decisions. In another study, Zhan et al. (2018) reported more negative N2 and positive LPP amplitudes when participants were making self‐sacrificing decisions for the benefit of strangers but not when making self‐sacrificing decisions for friends and acquaintances. Similar target‐specific differences were also found in the P3 component. For instance, P3 amplitudes were more positive when participants were deciding to help a friend versus a stranger gain monetary rewards (Li et al. 2020) and when participants were publicly versus privately making decisions that would benefit strangers and acquaintances (Li et al. 2022).

The FRN, P3, and N2 components were examined while participants evaluated helping outcomes. In two studies by Gan et al. (2016, 2022), FRN amplitude was more negative when participants received feedback that their decision to help resulted in a failed versus successful outcome. A more negative FRN amplitude (Gan et al. 2022) predicted participants' subsequent donation behaviors, including their willingness to donate money and smaller donations. Similarly, a more positive P3 amplitude and delayed latency were elicited when participants received feedback that their helping decision failed, which was also predictive of their subsequent willingness to donate and larger donations (Gan et al. 2022). In another study (Teng et al. 2018), P3 amplitudes were more positive when participants viewed scenarios that depicted a character needing help, but help was not offered by a third party. This study also examined the effects of a prosocial video game on N2 amplitude modulations and found participants who played the prosocial video game had the most negative N2 amplitudes when help was needed but not offered. On the contrary, the non‐prosocial video game group had the most negative N2 when help was needed and offered.

### 3.3 Multidimensional Characteristics of Prosocial Behaviors

Prosocial behaviors for each study were characterized according to the Heuristic Multidimensional Model of Prosocial Behaviors (Carlo and Padilla‐Walker 2020) and are included in our searchable Supporting Information database (SITable2.Task in Data S1). Studies did not present data regarding individuals' motives (e.g., selfish vs. selfless, intrinsic vs. extrinsic), so these characteristics were not considered in connection with ERP and EEG findings. Most studies (*n* = 26, 83.9%) recorded prosocial behaviors in non‐dire contexts (e.g., monetary pay‐off), two studies (6.5%) had a dire context (e.g., urgent need; Teng et al. 2018; Zhan et al. 2018), and three studies (9.7%) had insufficient information to distinguish this aspect of the context (Gan et al. 2016; Meidenbauer, Cowell, Killen, and Decety 2018). Most studies measured spontaneous prosocial behaviors (*n* = 29, 93.5%), though two studies (6.5%) focused on compliant behaviors (e.g., obeying a command; Kanso et al. 2014; Yang et al. 2022). Regarding emotional context, one study (3.2%) measured participants' self‐reported emotions during the paradigm (Zhan et al. 2018), two studies (6.5%) chose charities for the paradigm based on participants' personal empathy ratings for each charity (Carlson et al. 2016; Tan et al. 2025), and the remaining 28 studies (90.3%) did not provide relevant information. Most studies were set up to measure public prosocial behaviors (*n* = 27, 87.1%), while two studies (6.5%) attempted to give participants a sense of privacy (e.g., participant was in a private booth; Carlson et al. 2016; Cui et al. 2018) and two studies (6.5%) included a public and a private condition (Lavín et al. 2023; Li et al. 2022).

Regarding the targets of prosocial behaviors, most studies aimed to measure prosocial behaviors towards targets that were non‐kin and strangers (*n* = 26, 83.9%). Only five studies (16.1%) examined prosocial behaviors towards acquaintances and friends of participants (Kwak et al. 2020; Li et al. 2020, 2022; Chen et al. 2017; Zhan et al. 2018). Although almost half of the studies paired participants with same‐gendered partners or focused on same‐gendered friends (*n* = 14, 45.2%), studies did not explicitly report whether the targets of the prosocial behaviors were in‐group or out‐group members, so this target characteristic was not considered further.

### 3.4 Ecological Validity of Experimental Paradigms

**FIGURE 3:**
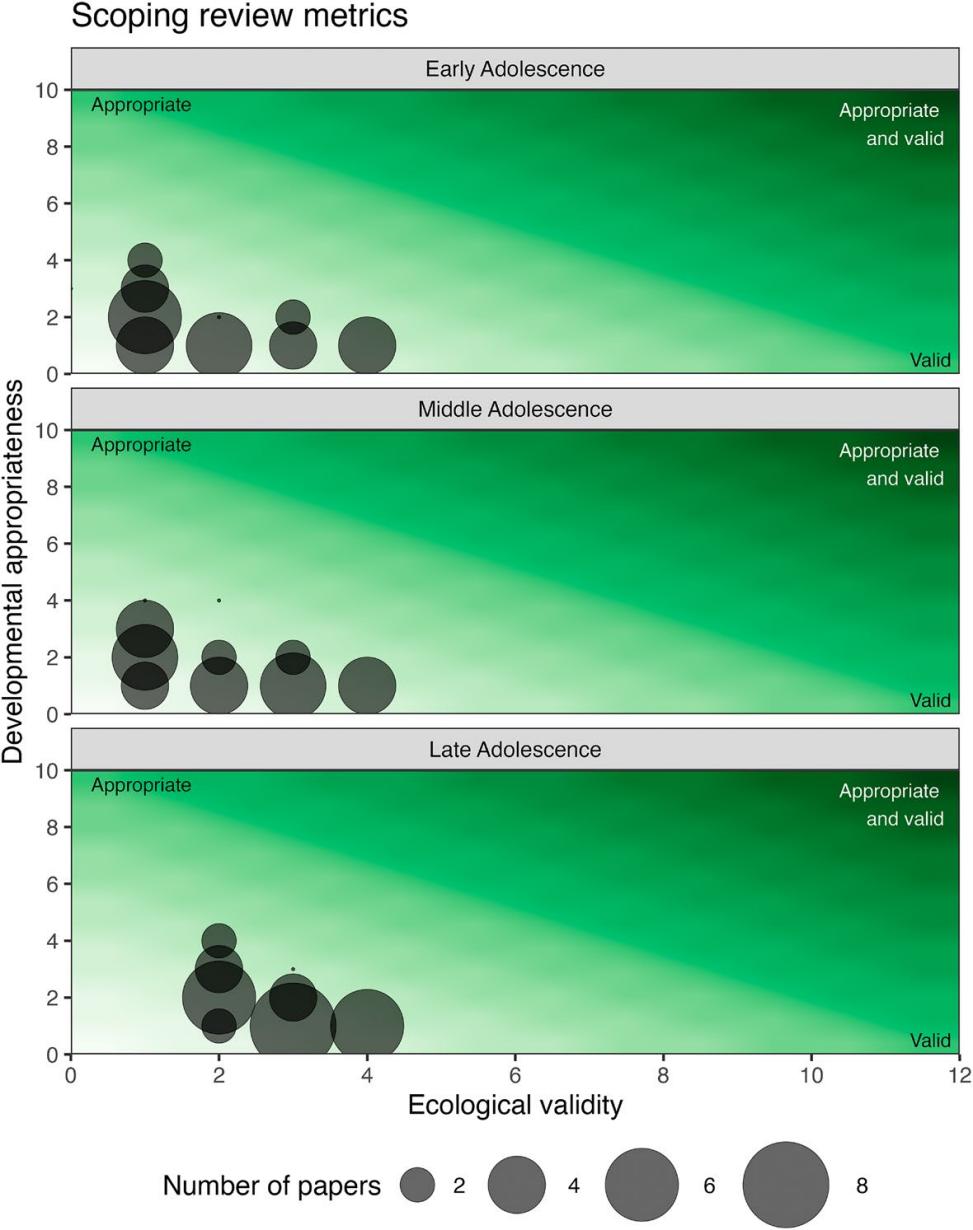
Ecological validity and developmental appropriateness overlap. For each developmental period, the diameter of the bubble represents the number of study paradigms at a particular developmental appropriateness and ecological validity value. The color gradient reflects the peak of high EV (*y* = 10) and high DA (*x* = 12).

Total scores and element‐level scores are provided in our searchable Supporting Information database (SITable3.EV in Data S1) and are depicted relative to developmental appropriateness (Section 3.5) in Figure 3. On ecological validity, most study paradigms scored in the low range (*n* = 29, 93.5%), with scores ranging between 0 and 3, and two studies scored in the moderate range (6.5%) with a score of 4. Although all study paradigms took place in a laboratory as opposed to real‐life settings, participants were often told their decisions would affect others, such as another participant or a charity (*n* = 22, 71%). In some paradigms, the participant was also introduced to another player, often a confederate, and would see them enter another room before they were led into their own room (*n* = 10, 32.3%). Only two study paradigms (6.5%) had participants complete the paradigm with a real participant (Li et al. 2020; Zhang et al. 2019) meaning their interactions involved an actual exchange with a non‐confederate. Seven studies were computer‐based without outcomes affecting others (22.6%).

Most study paradigms used unrealistic schematic visuals (e.g., simple shapes with numbers or words; *n* = 24, 77.4%), which represents the least ecologically valid stimuli. One study paradigm used semi‐realistic visuals (Ye et al. 2022), and five study paradigms used real‐life photos (Chen et al. 2017; Li, Li, et al. 2021; Pei et al. 2021; Yuan et al. 2019). All study paradigms used static stimuli versus dynamic stimuli. One study paradigm did not describe or provide images of their stimuli, so these features could not be evaluated (Meidenbauer, Cowell, Killen, and Decety 2018). Regarding the participant's response, all behavioral responses utilized a button press, which is typical of EEG/ERP studies, but is still considered less ecologically valid than behavioral responses that meet the demands of the real‐life situation. Lastly, regarding the phenomena measured, five study paradigms captured prosocial behaviors without reliance on monetary structure (Gan et al. 2016, 2022; Kanso et al. 2014; Meidenbauer, Cowell, Killen, and Decety 2018; Teng et al. 2018; Zhan et al. 2018), while the remaining paradigms relied on monetary structure (*n* = 26, 83.9%).

### 3.5 Developmental Appropriateness of Experimental Paradigms

Developmental appropriateness total scores ranged from 3 to 12, representing low (*n* = 1, 3.2%), moderate (*n* = 20, 64.5%), and high (*n* = 10, 32.3%). Total scores and element‐level scores are provided in our searchable Supporting Information database (SITable4.DA in Data S1) and are depicted relative to ecological validity (Section 3.4) in Figure 3. Most of the study paradigms (*n* = 28, 90.3%) were evaluated as feasible across the full range of adolescence. Two study paradigms (Zhan et al. 2018; Zhang et al. 2019) were deemed practical for only middle and late adolescence/emerging adulthood, and one paradigm (Teng et al. 2018) was deemed practical for only late adolescence/emerging adulthood. These study paradigms required more advanced skills, such as the ability to quickly read and comprehend scenarios presented on a screen for a limited time. More than half of study paradigms (*n* = 21, 67.7%) were regarded as not having relevant content (i.e., content reflecting the prosocial behaviors that adolescents may typically engage in on a regular basis). The 10 study paradigms (32.3%) that had more relevant content included charity donation paradigms (Carlson et al. 2016; Xu et al. 2023; Ye et al. 2022; Zhang et al. 2021), a resource allocation paradigm (Meidenbauer, Cowell, Killen, and Decety 2018), and helping paradigms (Gan et al. 2016, 2022; Li, Li, et al. 2021). Six study paradigms (19.4%) were regarded as more engaging across adolescent age ranges, while seven study paradigms (22.6%) were regarded as engaging for late adolescence/emerging adulthood only, and 17 study paradigms (54.8%) were regarded as less engaging across adolescence. Almost half of the study paradigms (*n* = 14, 45.2%) were regarded as easy to comprehend across adolescence whereas the other half may be more easily comprehensible to older adolescents.

## 4 Discussion

In this scoping review, we provided an overview of how EEG methods have been used to study the neural mechanisms of state‐level prosocial behaviors during early adolescence (10–13 years old), middle adolescence (14–17 years old), and late adolescence/emerging adulthood (18–25 years old). To fully characterize the methods used in this area, we systematically evaluated the ecological validity and developmental appropriateness of the experimental paradigms used in the reviewed studies. Applying a multidimensional framework of prosocial behaviors (Carlo and Padilla‐Walker 2020), we summarized EEG/ERP neural correlates identified across cooperation, fairness, giving, and helping paradigms.

Overall, our findings highlight an extreme lack of EEG/ERP research during younger and middle adolescence (10–17 years old), with only two studies examining prosocial behaviors in this age range (Kwak et al. 2020; Meidenbauer, Cowell, Killen, and Decety 2018) and the remaining studies focusing on college‐aged samples. Additionally, the reviewed studies were entirely cross‐sectional, limiting our ability to draw conclusions about developmental neural processes. As a result, it remains unclear whether changes in adolescents' neural systems precede, develop alongside, or result from changes in prosocial behavior. To fully understand how adolescents' changing neural systems relate to prosocial behavior, including directionality of these associations, longitudinal studies that assess within‐individual change over time are needed. While the scarcity of research across adolescence limits conclusions we can make about developmental EEG/ERP neural correlates, our characterization of methods and findings from older adolescent/emerging adult populations provides guidance for extending this work into earlier stages of adolescence.

### 4.1 EEG/ERP Neural Mechanisms: Broad Trends

Across reviewed studies and prosocial domains, P3 and FRN components were most frequently examined, highlighting a central role in prosocial processing. Consistent with the broader P3 literature (Rossi et al. 2017), P3 amplitude was sensitive to conditions with greater emotional or motivational salience, such as when viewing opponent cooperation (Cui et al. 2018), making decisions involving personal monetary reward or loss (Kwak et al. 2020), and benefitting friends (Li et al. 2020). P3 amplitude also predicted in‐person, state‐level prosocial behaviors, including donating (Carlson et al. 2016), resource allocation (Meidenbauer, Cowell, Killen, and Decety 2018), and helping (Gan et al. 2022). FRN findings were similarly consistent with the broader literature (Holroyd et al. 2009). FRN showed increased negativity when participants' outcome expectations were violated in helping (Gan et al. 2016, 2022), giving (Xu et al. 2023), cooperation (Chen et al. 2017), and fairness (Ma et al. 2015) paradigms. Together, these patterns suggest that the P3 and FRN reflect complementary neural processes involved in adaptive learning mechanisms that guide prosocial behavior.

Although the P3 and the FRN relate to similar neural processes across developmental stages, prior work demonstrates that these components are sensitive to developmental changes associated with neural maturation (Eppinger et al. 2009; Riggins and Scott 2020; van Dinteren et al. 2014). For example, one study found that children and adolescents had more positive P3 amplitudes when anticipating social and monetary rewards compared to adults (Wang et al. 2020), which may suggest heightened motivational salience during these developmental periods. Yet, notably in adolescents, faster behavioral responses in high social reward contexts were associated with shorter P3 latencies (Wang et al. 2020), possibly indicating more efficient processing in socially rewarding contexts. Although our review findings do not allow for definitive conclusions regarding developmental patterns due to the limited studies of younger adolescent samples, the existing evidence presented here further suggests that adolescence represents an important period of heightened sensitivity in social and motivational processing, and these relations warrant additional investigation.

Considering that adolescent fMRI work implicates reward‐responsive brain regions (e.g., nucleus accumbens, vmPFC) under similar conditions (Brandner et al. 2021), the P3 may correspond with underlying motivational processes that drive different prosocial behaviors in similar ways, particularly as major P3‐related brain networks undergo maturation. In contrast, the FRN is theorized to be generated in the anterior cingulate cortex (ACC) and related to reward prediction errors, which is critical in learning and behavior modification (Holroyd et al. 2009). Given its role in adolescent development (Blakemore 2008) and within contexts of empathy and social pain (Gu et al. 2010; Kawamoto et al. 2012), complementary investigations of the FRN could provide a deeper understanding of ACC regulation and modulation of prosocial expectancy (e.g., decoding the time course of ACC engagement).

### 4.2 EEG/ERP Neural Mechanisms: Developmental Trends

Several ERP components associated with prosociality in early childhood (Katus et al. 2020) were also examined in the reviewed studies: FRN, N2, P3, and LPP. Despite this overlap, direct developmental comparisons were not possible due to substantial differences in paradigm design. Paradigms used in infancy and early childhood often measured passive observation of unknown entities, whereas adolescent/emerging adult paradigms more often involved active decision making with consequences for oneself and others. Only similarities across fairness paradigms allowed for developmental comparison. In early childhood, the FRN is sensitive to unequal distributions (Cowell et al. 2019), and findings from the reviewed studies suggest that this sensitivity extends into late adolescence/emerging adulthood, including in regard to contextual factors. For example, FRN was modulated in late adolescence/emerging adulthood based on another person's intentions (Ma et al. 2015) and their financial capacity (Pei et al. 2021). Similarly, the LPP is sensitive to unequal resource allocations in both young children (Cowell et al. 2019) and 10–16‐year‐old adolescents (Meidenbauer, Cowell, Killen, and Decety 2018), and LPP responsivity was predictive of sharing behaviors across age groups. Thus, these findings suggest that the FRN and LPP may underlie fairness interpretations across childhood and adolescence with increased sensitivity to context in later ages, though this needs to be further examined.

In contrast, several EEG/ERP correlates have not been examined consistently across developmental stages. For example, the EPN and frontal alpha asymmetry have primarily been examined in infant and child studies (Cowell and Decety 2015a, 2015b; Crespo‐Llado et al. 2018; Decety et al. 2018), while the LFN, N1, P2, beta oscillations, and theta oscillations have primarily been examined in adolescent and emerging adult studies. To distinguish how these neural processes change across development as cognitive systems become more mature, future studies should prioritize measuring these components and power spectra across age groups, ideally by using complementary paradigms that bridge passive paradigms used in early development with active decision‐making paradigms used with older samples. These approaches will be critical for clarifying how neural systems supporting prosocial behaviors develop over time and for identifying potential neurobiological strengths associated with heightened social learning during adolescence.

### 4.3 Multidimensional Characteristics of Prosocial Behaviors

Organizing this review within the Heuristic Multidimensional Model of Prosocial Behaviors (Carlo and Padilla‐Walker 2020) highlighted how ERPs vary based on contextual and interpersonal factors. Across reviewed studies, ERP differences were evident depending on situational context (e.g., social comparison, private versus public settings) and target characteristics (e.g., social closeness, financial need). At the same time, this framework revealed gaps in how prosocial behaviors have been studied using EEG methods. One notable gap is the role of motivation, which is especially relevant to adolescent development. Although most studies clearly defined the context and targets of prosocial behaviors, few studies assessed participants' underlying motives. This is a critical limitation given that adolescence is characterized by changes in reward‐related neural systems (Cao et al. 2021), including heightened sensitivity to social reward (Blakemore and Mills 2014). Additionally, different motivations for engaging in prosocial behaviors are differentially associated with well‐being, such that more selfless motives relate to more positive outcomes (Davis et al. 2016). Indeed, prior work examining motives underlying prosocial behaviors in children and adolescents has called for more systematic examination of prosocial motives across development, individuals, and contexts (Eisenberg et al. 2016). Given the challenges of assessing motives subjectively through self‐report, neural techniques, such as EEG, may be especially useful for capturing implicit motivational processes that underlie prosocial behavior (Eisenberg et al. 2016).

Another gap was identified in the types of contexts that prosocial behaviors have been studied. Most studies examined prosocial behaviors in spontaneous, non‐dire, and public contexts, while relatively few focused on compliant, dire, or private contexts. Additionally, only one study included a self‐report measure of participants' emotional reactions, despite the high likelihood that multiple paradigms evoked strong emotional reactions. This is particularly relevant for adolescence because developmental changes in adolescence include heightened emotional reactivity (McLaughlin et al. 2015) and sensitivity to social evaluation (Blakemore and Mills 2014). Examining neural responses across emotional and non‐emotional contexts may therefore be especially important for understanding prosocial behavior during this period.

Finally, gaps in prosocial behavior targets emerged. While most studies focused on prosocial behaviors directed towards strangers and several studies included friends and acquaintances, no studies included family members. Findings suggest that neural responses and prosocial behaviors vary based on social distance (e.g., strangers compared with acquaintances). In comparison to social distance, other relational dimensions, like relationship quality remain largely unexplored. Future work should examine relationship quality in addition to social distance and extend this work to other important relationships, like family members. This work is particularly important based on fMRI evidence showing unique neural and behavioral patterns underlying adolescents' prosocial behaviors towards family members (Telzer et al. 2011).

### 4.4 Ecological Validity and Developmental Appropriateness

Increasing the ecological validity of neurocognitive paradigms can be complex and is not appropriate for every research objective; however, ecological validity is essential for understanding prosocial behaviors that generalize to daily life. Although most reviewed paradigms had low ecological validity, a few studies demonstrated how small adjustments can be made to increase the ecological validity of the study environment (e.g., dyadic interactions; Zhang et al. 2019) and stimulus (e.g., real‐life photos; Li, Li, et al. 2021). Similarly, some studies combined multiple methods to examine whether neural processes recorded during a less ecologically valid paradigm (e.g., computerized donation task) predict subsequent in‐person behaviors (e.g., donating; Gan et al. 2022). In fact, greater giving in economic games by adolescents has been linked with self‐reported online emotional support (van de Groep et al. 2026). When experimental paradigms demonstrate low ecological validity, linking neural processes evoked in these paradigms to everyday behaviors in comprehensive, multi‐method studies may inform generalizations towards meaningful outcomes (Berkman and Falk 2013).

Considering that adolescents' online social experiences are rapidly evolving (Armstrong‐Carter and Telzer 2021; Nesi et al. 2018) and online emotional support and activism are prominent forms of adolescent prosocial engagement (Armstrong‐Carter and Telzer 2021; Erreygers et al. 2018; van de Groep et al. 2026), it is important to understand whether the reviewed prosocial paradigms also meaningfully captured online forms of prosocial behavior. Despite all reviewed paradigms being computerized, only one attempted to emulate adolescents' online social environments (Xu et al. 2023). In contrast, other paradigms relied on abstract economic exchanges or hypothetical vignettes that do not reflect the social cues, relational contexts, or platform features adolescents encounter offline (e.g., interacting with familiar peers) or online (e.g., social media feeds, public versus private responses). Ecologically valid paradigms that emulate adolescents' online social environments while accommodating EEG methodological constraints are needed. Moving forward, future studies could adapt paradigms to incorporate naturalistic elements such as video stimuli of real peers, recruitment of friend dyads, simulated social media interfaces with visible social feedback, opportunities to compose supportive messages, and meaningful real‐world consequences (e.g., actual donations that affect real people). Additional inspiration can be drawn from fMRI studies that have successfully emulated adolescents' online social environments (Silk et al. 2012) as well as findings from self‐report studies that describe the online prosocial behavioral patterns of adolescents (Erreygers et al. 2018).

In evaluating developmental appropriateness, the reviewed paradigms often reflected activities that adolescents are unlikely to perform in their daily life, and paradigms appeared more appropriate for older adolescents in terms of potential engagement and ease of comprehension than younger to middle‐aged adolescents. Given that most studies focused on college‐aged samples, it is understandable that the experimental paradigms used in these studies were not purposely designed to be developmentally appropriate for the full range of adolescence. To meet the developmental needs of younger adolescents, it is possible to adapt existing paradigms or create new paradigms. As previously noted, incorporating video stimuli of real peers or recruiting friend dyads may be particularly helpful in making prosocial paradigms, particularly those that involve abstract economic decisions or hypothetical situations, more developmentally accessible for younger adolescents. Indeed, hyperscanning approaches have proven useful for capturing neural correlates of more dynamic and interactive aspects of social behavior (e.g., collaboration), including in adolescent and neurodiverse populations (Nelson et al. 2025), and may offer a promising avenue for studying prosocial processes in more naturalistic contexts. Similarly, the gamification of cognitive tasks (e.g., working memory paradigms) has been shown to enhance participant engagement without altering physiological measurements (Scharinger et al. 2023). Applying similar gamified elements to prosocial EEG tasks may therefore increase engagement and developmental appropriateness, particularly for early adolescence, while preserving measurement integrity. Finally, special consideration should be made to create ecologically valid paradigms that assess prosocial behaviors that adolescents deem important, for instance, as found in a qualitative study with adolescents (Bergin et al. 2003): standing up for others, helping others develop skills, complimenting and encouraging others, including others, and providing emotional support.

### 4.5 Limitations of Reviewed Studies

A primary limitation of the current scoping review is the small number of EEG/ERP studies with adolescents aged 10–17 years old, which were also cross‐sectional. This constrains our ability to characterize neural correlates of prosocial behaviors across the full span of adolescence and understand how mechanisms change across development. Still, the two studies that included younger adolescents, along with the studies of older adolescents/emerging adults, allowed us to characterize how EEG approaches have been applied to examine prosocial behaviors during adolescence and emerging adulthood. These findings provide directions for future EEG/ERP studies, particularly in younger to middle adolescence.

Similarly, it is important to note that our scoping review is limited to the data that was reported in the reviewed studies, and our findings may be most generalizable to similar samples. Specifically, 26 studies of the 31 studies included in this scoping review focused on college student samples in China. Considering the role that culture and context have in shaping prosociality (Carlo and Padilla‐Walker 2020), measures of cultural identity and/or cultural values will be important to include in future EEG/ERP studies to capture how cultural factors may uniquely influence the rapid cognitive processes underlying adolescent and emerging adult prosocial behaviors.

Another limitation is that many of the reviewed studies overly relied on a monetary decision‐making structure to examine prosocial behaviors, likely involving different cognitive processes than typical day‐to‐day prosocial decision‐making, especially for adolescents, as noted in prior fMRI reviews (Crone et al. 2022; Sipes et al. 2022). Further, a study in the current review found ERP differences between monetary and non‐monetary conditions (Li, Xu, and Zhong 2021). Despite most studies relying on a monetary structure, only one study measured participants' household income (Meidenbauer, Cowell, Killen, and Decety 2018). This is an issue given previous work demonstrating that socioeconomic status can influence prosocial behaviors across development (Miller et al. 2015; Schulreich et al. 2023). Future studies could assess the economic background of participants and carefully consider how this and how other demographic information may be pertinent and potentially influence or motivate prosocial behaviors, particularly when measured by monetary tasks. Additionally, there a need for the creation and use of tasks in cognitive neuroscience that assess prosocial behaviors without relying on monetary allocation.

To our knowledge, we provided the first attempt at creating criteria to evaluate the developmental appropriateness of cognitive neuroscience paradigms. While more work is needed to further develop and validate the scoring procedures and rubric we created, it is critical to have a systematic method of evaluating and creating paradigms with consideration of target populations' developmental and clinical needs. This will ensure better measurement and understanding of study constructs as well as increase the generalization of cognitive neuroscience findings. Our scale had coding inconsistencies (e.g., feasibility for early adolescence) and weak reliability (e.g., engagement potential for late adolescence/emerging adulthood), which may be related to highly imbalanced marginal distributions (e.g., numerous “0” codes). We recommend that the coding scale be refined beyond a binary scale to better account for nuances. In addition, it is critical to receive feedback from the target population on this new rating system—we also recommend having adolescents provide feedback on how the coding scale is used and what criterion should be considered.

Our ecological validity rating system could also be strengthened for future use. Based on the three dimensions of ecological validity proposed by Schmuckler (2001), this rating system was designed to assess how accurately cognitive neuroscience paradigms reflected the real‐world prosocial behavior that each study was aiming to measure. A fourth dimension, phenomena measured, was added to assess the tendency for EEG cognitive neuroscience paradigms t use a monetary decision‐making structure to assess prosocial behaviors. While this was important to assess in the current scoping review, as we identified 83.9% of study paradigms used a monetary decision‐making structure, we are aware that prosocial behaviors can occur within a monetary decision‐making context, and this is ecologically valid if it aligns with the aims of the study. Accordingly, future iterations of this rating system could adjust the definition of Phenomena Measured, possibly pulling from Content Relevance in the developmental appropriateness rating scale (“Is it likely the participant will do this activity (as related to the phenomenon being measured) in their daily life?”). For instance, the Phenomena Measured criterion could focus on how well the activity being performed matches the real‐life phenomenon the study is aiming to assess.

### 4.6 Implications for Promoting Prosocial Behavior Tendencies in Adolescence

Adolescence is proposed to be a sensitive period for sociocultural processing due to the neurobiological maturation of affective and advanced social cognitive systems and heightened experience‐dependent plasticity, which likely underlies the development of prosocial behaviors (Baker et al. 2025; Cheng et al. 2024; Crone and Dahl 2012; Larsen and Luna 2018). EEG methods offer temporal and developmental advantages for characterizing rapid, implicit neurocognitive mechanisms that evolve as neural systems mature and cannot be fully captured through self‐report or behavioral measures (Hudac and Sommerville 2020; Johnson and de Haan 2015). Leveraging a combination of these methods during adolescence can help identify neurocognitive strengths associated with heightened sociocultural processing and learning, as well as mechanisms that may be particularly responsive to environmental input. Characterizing these developing neural processes, especially in terms of adaptive social learning and motivation, may provide a unique opportunity to inform efforts to promote prosocial behavior patterns that persist into adulthood. Further, understanding these neurocognitive mechanisms may clarify mechanistic differences underlying reduced prosociality across various psychological disorders. This knowledge could guide the optimization of interventions based on individual and developmental needs and inform the intentional shaping of natural environments, including relational contexts, to support prosocial developmental trajectories.

## 5 Conclusions

This scoping review characterized and evaluated EEG/ERP research on state‐level prosocial behaviors in adolescence and emerging adulthood. Across 31 reviewed studies, sample characteristics were geographically and developmentally restricted, primarily focusing on college‐student samples from China. Notably, very few studies included early or middle adolescents or conducted age‐related comparisons, highlighting a significant gap in the use of EEG/ERP methods across the full span of adolescence. In addition, prosocial behaviors were primarily examined within cooperation, fairness, giving, and helping paradigms, many of which were deemed less developmentally appropriate for younger adolescents. Future work should prioritize developing ecologically valid prosocial behavior paradigms that capture behaviors more typical of younger adolescents' daily lives, such as everyday forms of helping, providing emotional support to peers, and engaging in self‐regulation to benefit others.

Despite variability in paradigms, trends emerged in P3 and FRN components across prosocial domains. P3 amplitude was consistently sensitive to emotional/motivational salience and was predictive of real‐world, state‐level prosocial behaviors across giving, fairness, and helping domains. FRN amplitude showed consistent increases negatively when outcome expectations were violated across all prosocial domains. Together, these findings suggest that the P3 and FRN are key neural mechanisms underlying adaptive learning across prosocial contexts. To clarify how these components relate to heightened sensitivity in social learning proposed to characterize adolescence, future research should examine these components across the full range of adolescence.

Finally, by organizing the studies within a multidimensional framework of prosocial behaviors, this review demonstrated how ERPs are sensitive to the context and targets of prosocial behaviors, which emphasizes the importance of considering the multidimensional nature of prosocial behaviors in study design and interpretation of findings. Importantly, the motives underlying prosocial behaviors remain a critical area to be examined, particularly in relation to adolescents' developing neural systems. EEG/ERP methods are especially well positioned to evaluate fast‐acting, implicit motivational mechanisms.

## Author Contributions

**Kate Flory:** writing – review and editing. **Caitlin M. Hudac:** writing – review and editing, visualization, supervision. **Abigail L. Hogan:** writing – review and editing. **Sarah R. Edmunds:** writing – review and editing. **Cailee M. Nelson:** validation, writing – review and editing. **Rebecca Revilla:** conceptualization, investigation, writing – original draft, methodology, writing – review and editing, visualization, data curation, formal analysis.

## Funding

The authors have nothing to report.

## Ethics Statement

This study is a review of previously published literature and did not require ethical approval from an institutional review board (IRB).

## Conflicts of Interest

The authors declare no conflicts of interest.

## Supporting information

A searchable database **(Data S1)** is provided and includes
**SI Table1.** Summary of ERP/EEG results.
**SI Table2.** Task details and multidimensional characteristics.
**SI Table3.** Ecological validity ratings.
**SI Table4.** Developmental appropriateness ratings.

Supplemental figures **(Data S2)** include
**SI Figure 1.** Ecological validity inter‐rater reliability.
**SI Figure 2.** Developmental appropriateness inter‐rater reliability.
**SI Figure 3.** Ecological validity rating system.
**SI Figure 4.** Developmental appropriateness rating system.

## Data Availability

We include a fully searchable database of reviewed study findings in the Supporting Information of this article.
