# Supplementary material for: Neural Foundations Supporting Prosocial Behaviors: A Scoping Review of EEG/ERP Evidence During Adolescence and Emerging Adulthood
Source: Psychophysiology. 2026 Jun 18;63(6):e70340. doi: 10.1111/psyp.70340 (PMC13277634; doi:10.1111/psyp.70340)
Supplement: Supplementary file 2 — Supplemental figures (Data S2) include SI Figure 1. Ecological validity inter‐rater reliability. SI Figure 2. Developmental appropriateness inter‐rater reliability. SI Figure 3. Ecological validity rating system. SI Figure 4. Developmental appropriateness rating system. [file PSYP-63-e70340-s001.docx]

**Supplemental Information Figures.**

**Coding reliability.** Coders discussed their independent scoring and amended discrepant scores after discussion. To assess inter-rater reliability across independent coders, percent agreement across coders and weighted Cohen’s kappa was calculated using the kappa2 function within the *irr* R package. Weighted Cohen’s kappa was chosen to assess interrater reliability based on our scoring system’s ordinal structure (Cohen, 1968).

**SI Figure 1. Ecological validity inter-rater agreement.** Inter-rater agreement percentage across studies was high (>70%) in all categories. Inter-rater reliability based upon weighted Cohen’s kappa was moderate (i.e., 0.41-0.60) to high (i.e., 0.81-1) across all criteria. Weighted Cohen’s kappa could not be estimated for Response because there was zero variance.


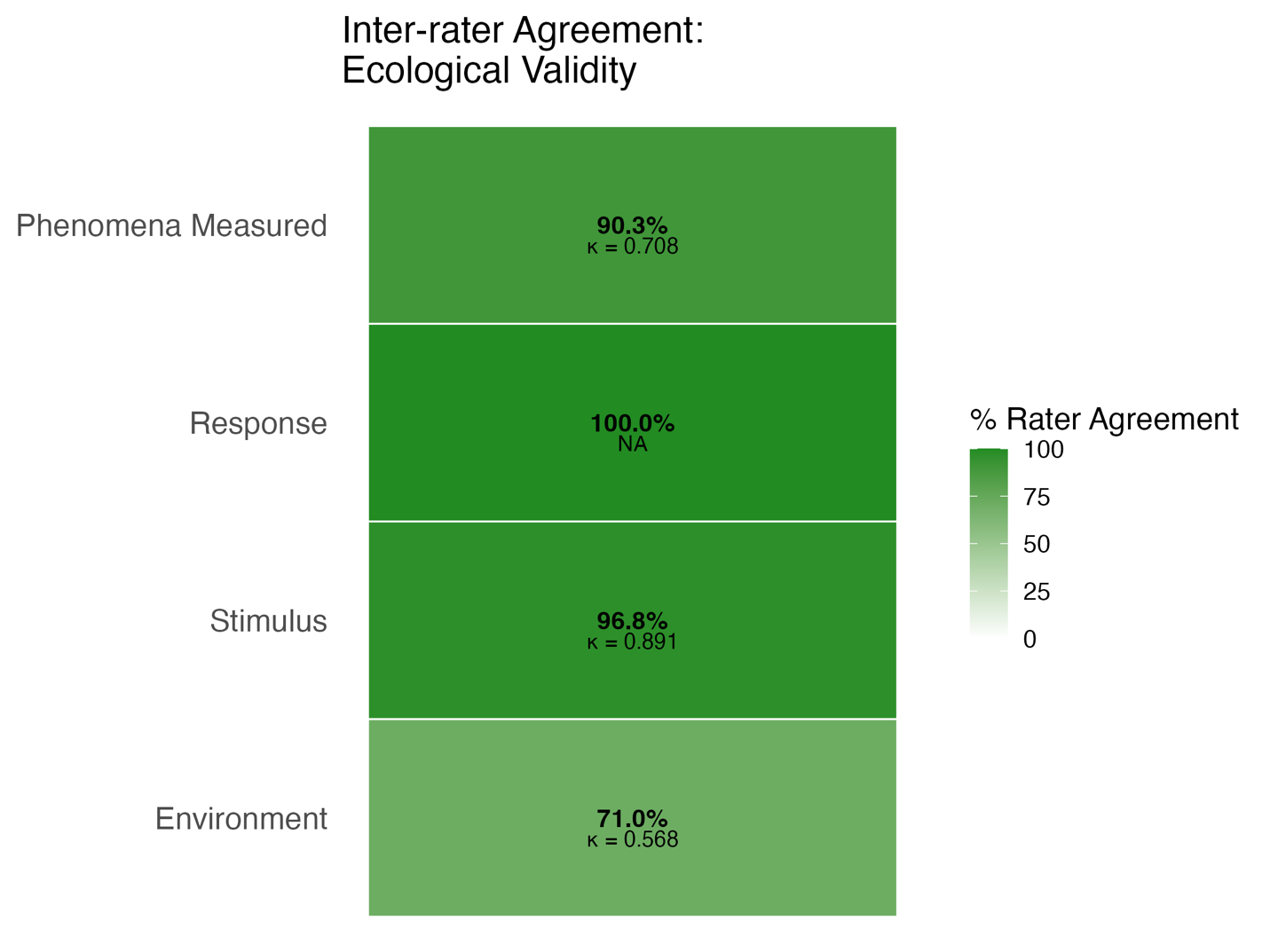


**SI Figure 2. Developmental appropriateness inter-rater reliability.** Inter-rater reliability was computed for each developmental stage: early adolescence, middle adolescence, late adolescence / emerging adulthood. Overall, percent agreement was fair-to-moderate (60-75%) to high (>75%) agreement across raters. In some cases, percent agreement was high (93.5%), but κ was low due to highly imbalanced marginal distributions, which may be addressed in the future by refining the coding scale to reduce extreme prevalence. To better validate in the current study, Cohen’s κ was supplemented with Gwet’s AC1 (Wongpakaran et al., 2013; calculated using the *irrCAC* R package), which is less sensitive to prevalence imbalance. AC1 values indicated substantial agreement across all criteria (fair = .21-.4, moderate = .41-.60, substantial > .61).

- **Moderate reliability concerns**: There was fair to moderate inter-rater reliability for Engagement Potential for early and middle adolescence (agreement 61–65%, κ ≈ 0.15–0.27, AC1 ≈ 0.31). There was moderate inter-rater reliability for late content relevance and middle ease of comprehension.
- **Low reliability concerns**: Criterion with low kappa and AC1 indicate a reliability concern beyond a prevalence artifact for the following:
  - **Early – Feasibility:** Agreement is below 50% and both kappa and AC1 are negative, which indicate a coding inconsistency.
  - **Late – Engagement potential:** Agreement is below 50% and both kappa and AC1 are small values, which indicate weak reliability.

**
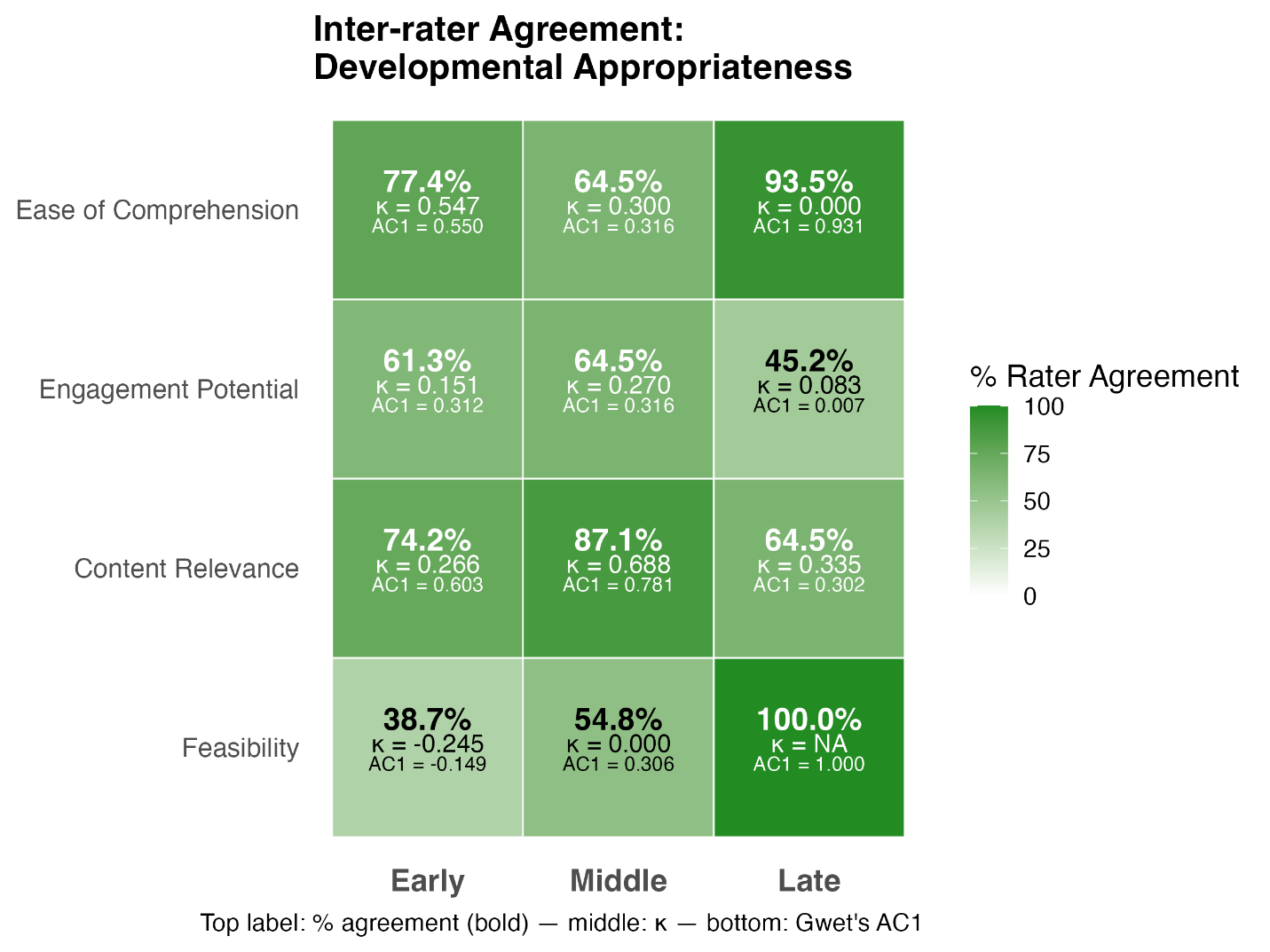
**

**SI Figure 3. Ecological validity rating system**. Based in part from Schmuckler 2001, each dimension was scored for each definition with some elements having multiple scoring criteria. Studies were given a total rating score of high (8-11), moderate (4-7), or low (0-3).

| **Ecological Validity Dimension** | **Range** | **Definition** | **Scoring criteria (Score)** |
| --- | --- | --- | --- |
| **Environment** | 0-4 | What are the features of the setting in which the study is being conducted? How closely does the setting reflect the environment the phenomenon naturally occurs in? | 1. Laboratory (0) **or** real-life setting (1) 2. Interactions occur with a computer/no acknowledgement of real person or entity (e.g., charity) (0) **or** participant is told decisions will affect another person or entity (1) participant is told decisions will affect another person or entity AND meet or see person/entity in person or in photos (2) **or** spontaneous interactions with a real person in real time or online (3) |
| **Stimulus** | 0-5 | What is the visual presentation of the target activity? How closely does the visual presentation of the target activity reflect real-world phenomena? | 1. Unrealistic schematic visuals (0) **or** semi-realistic visuals (e.g., cartoon images) (1) **or** real-life photos (2) **or** virtual reality (3) **or** real-life scenario (4) 2. Static stimuli (0) **or** dynamic/animated stimuli (1) |
| **Response** | 0-1 | What is the method of recording behaviors in response to the target activity? How closely does this response behavior reflect natural responses to the target phenomenon? | 1. Button-press/mouse-click (0) **or** behavioral response that matches a response method that would typically meet the demands of the real-life situation (e.g., passively viewing images does not require a necessary response in real-life and would be scored a one) (1) |
| **Phenomena Measured** | 0-1 | Does the target activity capture prosocial behaviors without reliance on monetary structure? | 1. Relies on monetary structure (0) **or** captures prosocial behaviors without reliance on monetary structure (1) |

**SI Figure 4. Developmental appropriateness rating system.** Each dimension was scored for each developmental stage (early adolescence, 10-13 years old; middle adolescence, 14-17 years old; late adolescence/emerging adulthood, 18-25 years old). Studies were given a total rating scores of high (8-12), moderate (4-7), or low (0-3).

| **Developmental Appropriateness Dimension** | **Range** | **Definition** (0 = no, 1 = yes; One per developmental stage) |
| --- | --- | --- |
| **Feasibility** | 0-3 | Is it likely that the target population will possess the skills (e.g., motor, cognitive) required to successfully complete the target activity? |
| **Content Relevance** | 0-3 | Is it likely the participant will do this activity (as related to the phenomenon being measured) in their daily life? |
| **Potential Engagement** | 0-3 | Is this activity likely to interest or engage the participant? |
| **Ease of Comprehension** | 0-3 | Is this activity designed in a way (e.g., instructions, clarity of goal) that the target population would understand how to complete it? |

**References used in Supplemental Information**

Cohen, J. (1968). Weighted kappa: Nominal scale agreement provision for scaled disagreement or partial credit. *Psychological Bulletin, 70*(4), 213–220. https://doi.org/10.1037/h0026256

Schmuckler, M. A. (2001). What is ecological validity? A Dimensional analysis. *Infancy*, *2*(4), 419–436. [https://doi.org/10.1207/S15327078IN0204_02](https://doi.org/https:/doi.org/10.1207/S15327078IN0204_02)

Wongpakaran, N., Wongpakaran, T., Wedding, D., & Gwet, K. L. (2013). A comparison of Cohen’s Kappa and Gwet’s AC1 when calculating inter-rater reliability coefficients: A study conducted with personality disorder samples. *BMC Medical Research Methodology*, *13*(1), 61. https://doi.org/10.1186/1471-2288-13-61
